# Use of preoperative swept-source optical coherence tomography in vitrectomy for advanced diabetic eye disease: a pilot study

**DOI:** 10.3389/fopht.2026.1688660

**Published:** 2026-04-13

**Authors:** Arshad Mehmood, Altamash Shahriyar Ghazanfar, M. A. Rehman Siddiqui

**Affiliations:** 1 Shahzad Eye Hospital, Karachi, Pakistan; 2 Department of Ophthalmology and Visual Sciences, Aga Khan University Hospital, Karachi, Pakistan; 3 Retina & Eyecare Centre, Karachi, Pakistan

**Keywords:** diabetes mellitus, diabetic retinopathy, imaging, retina, vitreoretinal surgery

## Abstract

**Background:**

Swept-source optical coherence tomography (SS-OCT) is an OCT variant with longer wavelengths than other commonly used OCT devices. Our aim was to determine the benefit of pre-operative SS-OCT to detect occult retinal pathologies in patients with advanced diabetic eye disease (ADED).

**Methods:**

Fundus photos and SS-OCT were performed pre-operatively in all patients scheduled for vitrectomy for ADED. These were reviewed by two retina specialists. A modified BIO-score based on the Nussenblatt scale of vitreous haze was used to evaluate fundus clarity on fundus photos. Findings on fundus photos were compared with the findings on SS-OCT. Any disagreement between the retina specialists was resolved upon discussion.

**Results:**

We included 38 consecutive eyes of 35 patients. Various degrees of cataract was present in 22 eyes. In our sample, 84.2% of cases had a BIO score of 3 or more and 31.6% had a BIO score of 4 or 5. The most common diagnosis on fundus photos was vitreous haemorrhage (n = 23). Tractional retinal detachment (n = 20) was the most common diagnosis on SS-OCT. Only 3 cases had no view on SS-OCT. There was almost perfect (κ = 0.933, 95% CI: 0.887-0.979), substantial (κ = 0.734, 95% CI: 0.632-0.836) and moderate (κ = 0.593, 95% CI: 0.494-0.692) intergrader agreement for fundus photo diagnosis, SS-OCT diagnosis and BIO scores respectively.

**Conclusion:**

SS-OCT showed potential as an adjunct to clinical examinations, especially when hazy media opacities did not allow for a clear view of the fundus. However, it is important to recognise that OCT only serves as an adjunct and cannot be used as a definitive argument for assessing surgical feasibility.

## 1 Introduction

Diabetic retinopathy (DR) is the leading cause of blindness and visual impairment in working-age adults (1). Diabetic maculopathy and complications of DR such as vitreous haemorrhage (VH) and tractional retinal detachment (TRD) are often responsible for vision loss. Current modalities for treating these complications include pan-retinal photocoagulation (PRP), intravitreal anti-vascular endothelial growth factor (VEGF) injections and vitrectomy. Indications for vitrectomy for advanced diabetic eye disease (ADED) include non-clearing VH, TRD involving or threatening the macula, combined tractional and rhegmatogenous retinal detachment (CTRRD), and progressive fibrovascular proliferation (FVP) (2).

Optical coherence tomography (OCT) is a non-invasive imaging modality to evaluate retinal pathologies. Swept-source OCT (SS-OCT) is a Fourier-domain OCT variant. It generally uses a broadband swept source whose light source wavelength varies with time (3). Commercially available SS-OCT devices have often been associated with longer wavelengths (> 1000 nm) than other commonly used OCT devices (800–900 nm). A longer wavelength is important for improved visualisation of deeper ocular structures in the presence of media opacities as it is less scattered and penetrates deeper (4). SS-OCT has previously been used as a screening tool for detection of retinal pathologies in patients undergoing cataract surgery (5). It has also been shown to enable clearer visualisation of the posterior vitreous, allowing for better identification of disorders of the vitreomacular interface (6, 7).

Despite developments in surgical equipment and techniques, vitrectomy for ADED is still a challenging procedure, even for the most experienced surgeons. The role of SS-OCT for detection of retinal pathology in patients undergoing vitrectomy for ADED is not known. Many of these patients have VH, which complicates slit lamp fundus examination. In addition, patients with extensive FVP have membranes which hinder the view of underlying retinal layers. Therefore, a comprehensive pre-operative evaluation is crucial to plan the best possible surgical strategy and predict treatment outcomes.

The aim of this study is to assess the performance of pre-operative SS-OCT in patients undergoing vitrectomy for ADED.

## 2 Methods

This prospective cohort study was performed at Shahzad Eye Hospital, Karachi, Pakistan from January 2017 to August 2022. The study was approved by the hospital’s institutional review board and adheres to the tenets of the Declaration of Helsinki. Informed consent was taken from all included patients.

The inclusion criteria for our study was adult (>18 years) patients undergoing vitrectomy for ADED, who were able and willing to provide consent. ADED was defined as patients with VH, TRD, CTRRD and progressive FVP. Our exclusion criteria entailed the presence of glaucoma or optic atrophy pre-operatively.

The OpenEpi sample size calculator was used to determine the required sample size for our study. We estimated the prevalence of vitrectomy for ADED to be 5.7% among patients with proliferative DR (PDR). This was based on a study conducted in the UK due to the absence of local data (8). We set a margin of error of 5.5% at a 95% confidence interval. The estimated sample size using these parameters was found to be 69 patients.

**Figure 1:**
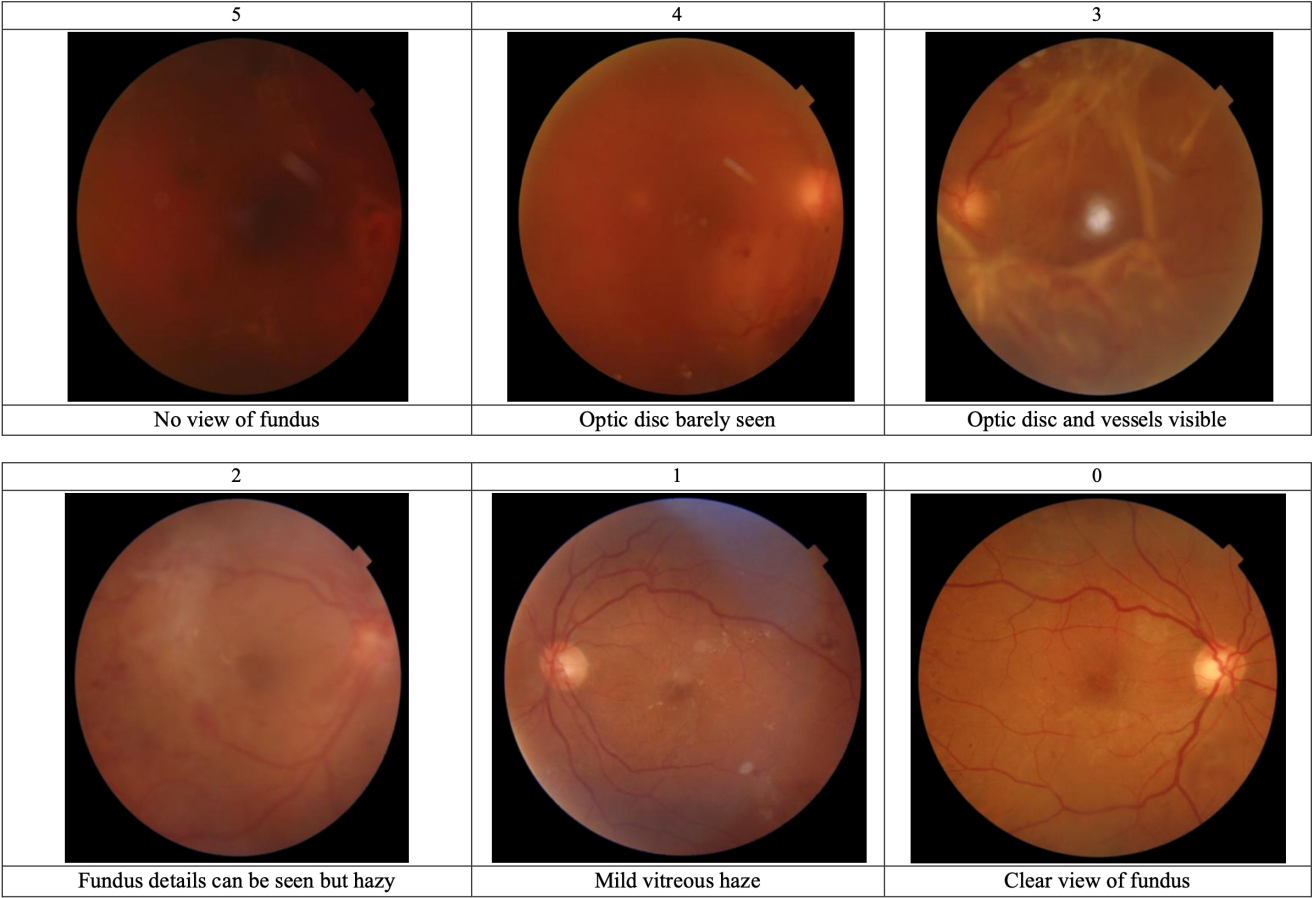
Modified BIO score descriptions and sample images. The modified BIO score was based on the Nussenblatt scale of vitreous haze.

Best corrected visual acuity (BCVA), applanation tonometry, slit lamp examination and dilated fundus examination was done for each patient pre- and post-operatively. BCVA was assessed using a Snellen chart and later converted to logMAR (9, 10). Data regarding the patients age, gender, duration of diabetes and history of cataract surgery was collected. Fundus photos (45° macula centred) and SS-OCT were performed by the same experienced OCT technician using the same machine (Triton, Topcon Healthcare, Japan) during the week preceding the surgery. The Triton SS-OCT machine uses a 100 kHz scanning speed and a wavelength of 1050 nm. Two retina specialists reviewed the fundus photos to make a diagnosis. Vitreous haze scoring was done by both retina specialists on each fundus photo according to a modified BIO score of vitreous haze (**Figure 1**). This score was based on the Nussenblatt scale of vitreous haze (11). Both specialists were masked to patient details and OCT findings when labelling the fundus photos. Lastly, single line SS-OCTs were evaluated by the same retina specialists for potential findings. Any disagreement between the retina specialists was resolved upon discussion.

Data analysis was performed using SPSS Statistics for MacOS, Version 23.0 (IBM, USA). Continuous variables were expressed as mean ± standard deviation and categorical variables were expressed as frequencies and percentages. Normality of data was assessed using the Shapiro-Wilk test. A paired t-test was used to compare the pre-operative BCVA with the BCVA 4 weeks after the procedure. As post-operative BCVA was not normally distributed, the Wilcoxon signed rank test was used to compare pre- and post-operative BCVA among patients who were already pseudophakic. Inter-grader agreement was assessed using Cohen’s kappa (κ). As per the Landis and Koch scale, interpretation of κ was as follows: ≤ 0.20 (slight agreement), 0.21-0.40 (fair agreement), 0.41-0.60 (moderate agreement), 0.61-0.80 (substantial agreement) and 0.81-100 (almost perfect agreement) (12).

## 3 Results

Our study included 38 eyes of 35 patients. The sociodemographic characteristics of the study population are displayed in **Table 1**. Among the 38 included eyes, 16 (42.1%) were pseudophakic. The remaining eyes had various degrees of cataract formation, so a combined phacoemulsification and vitrectomy procedure was planned in these cases. The overall mean pre-operative BCVA was 1.23 ± 0.68 logMAR, which improved to 0.90 ± 0.63 logMAR (p = 0.029) 4 weeks post-vitrectomy. Among patients who were pseudophakic pre-operatively, mean BCVA improved from 1.47 ± 0.68 logMAR to 0.86 ± 0.68 logMAR (p = 0.009).

**Table 1:** Sociodemographic characteristics of the study population (n = 35 patients).

Characteristic	n (%)/mean ± SD
Age (years)	53.4 ± 8.0
Gender	
Male	19 (54.3)
Female	16 (45.7)
Duration of Diabetes (years)	15.8 ± 7.1

**Table 2** compares the diagnosis on fundus photos with those after reviewing SS-OCT. On the basis of fundus photos, the most common primary diagnosis was VH, which was seen in 23 (60.5%) cases. TRD was diagnosed in 10 (26.3%) cases. Epiretinal membranes (ERM) were also noted in addition to the primary diagnosis in 2 (5.3%) cases. The most common diagnosis on SS-OCT was TRD, which was seen in 20 (52.6%) cases. ERMs were seen in 4 (10.5%) cases and in 4 (10.5%) cases the retina below the vitreous haemorrhage was determined to be normal.

**Table 2:** Comparison of the diagnosis on fundus photos with SS-OCT (n = 38 eyes).

Diagnosis	Fundus photos n (%)	SS-OCT n (%)
Vitreous Haemorrhage	23 (60.5)	4 (10.5)
Tractional Retinal Detachment	10 (26.3)	20 (52.6)
Fibrotic Bands	4 (10.5)	3 (7.9)
Proliferative Diabetic Retinopathy	1 (2.6)	0 (0)
Diabetic Macular Edema	0 (0)	4 (10.5)
Subhyaloid Haemorrhage	0 (0)	3 (7.9)
Macular Hole	0 (0)	1 (2.6)
No view on SS-OCT	–	3 (7.9)

Images were reviewed by 2 retina specialists, and any disagreement was resolved on discussion.

**Figure 2:**
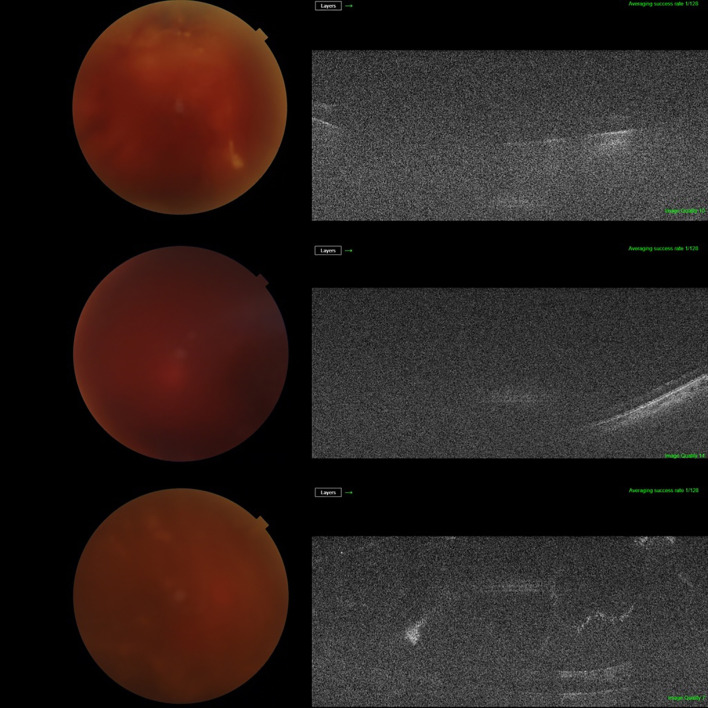
Cases with a modified BIO score of 5, where there was no view on SS-OCT.

**Figure 3:**
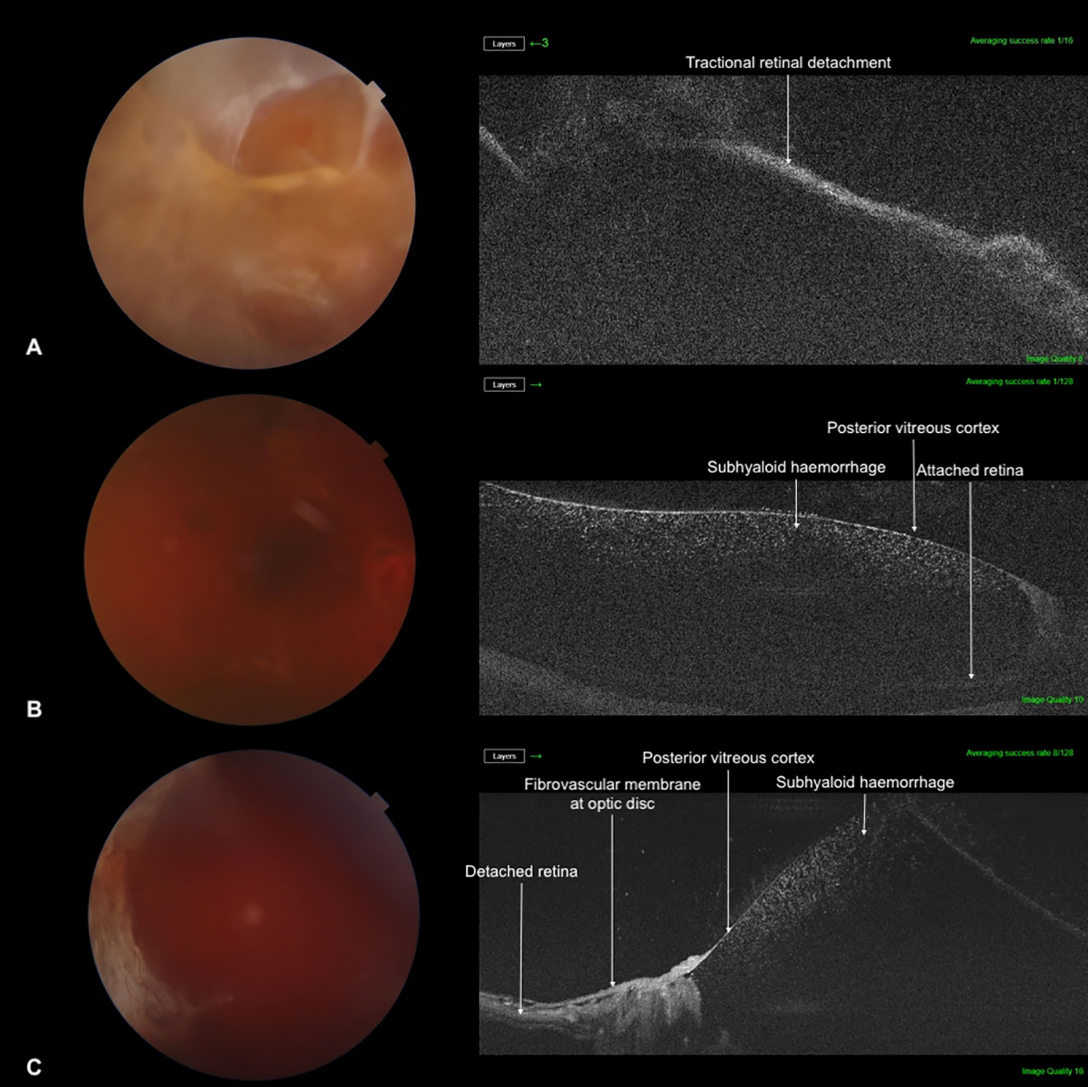
Remaining cases with a modified BIO score of 5, where SS-OCT resulted in a diagnosis. **(A)** Tractional retinal detachment **(B)** Subhyaloid haemorrhage **(C)** Subhyaloid haemorrhage.

**Figure 4:**
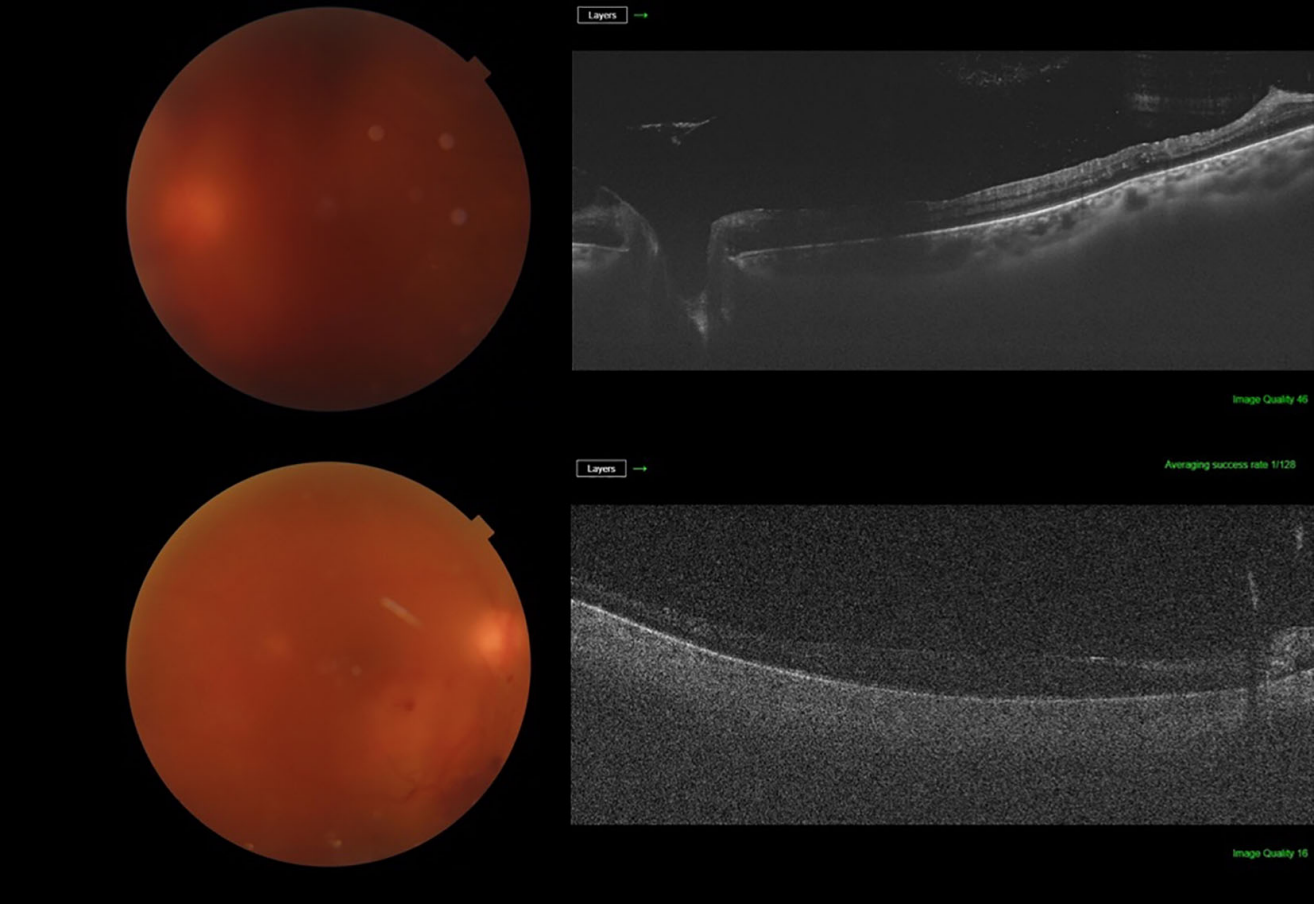
Cases where the modified BIO score was 4 and SS-OCT determined that the underlying retina was normal.

The distribution of BIO scores is shown in **Table 3**. In our sample, 84.2% of cases had a BIO score of 3 or more and 31.6% had a BIO score of 4 or 5. A BIO score of 5 was assigned to 6 cases, of which 3 had an unsatisfactory view of the retina on SS-OCT (**Figure 2**). Of the remaining 3 cases, 2 had a diagnosis of subhyaloid haemorrhage and 1 was diagnosed with TRD on SS-OCT (**Figure 3**). Among the patients who had a BIO score of 4, SS-OCT revealed that 2 had a normal retina (**Figure 4**) while TRD, subhyaloid haemorrhage and fibrotic bands were seen in 2, 1 and 1 cases respectively. A BIO score of 3 was seen in 20 (52.6%) cases. TRD was seen in 14 of these cases and 2 had a normal retina. Fibrotic bands, diabetic macular edema (DME) and ERMs were seen in the other 4 cases.

**Table 3:** Number of patients assigned each modified BIO score of vitreous haze (n = 38 eyes).

BIO score	n (%)
5	6 (15.8)
4	6 (15.8)
3	20 (52.6)
2	4 (10.5)
1	2 (5.3)
0	0 (0)

This score was based on the Nussenblatt scale of vitreous haze.

The intergrader agreement was highest for SS-OCT diagnosis, followed by fundus photo diagnosis and BIO score. There was almost perfect agreement (κ = 0.933, 95% CI: 0.887-0.979, p < 0.01) for SS-OCT diagnosis as both graders only disagreed on 2 cases. The graders disagreed on 6 cases for diagnosis based on fundus photos, resulting in substantial agreement (κ = 0.734, 95% CI: 0.632-0.836, p < 0.01). BIO scores had only moderate agreement (κ = 0.593, 95% CI: 0.494-0.692, p < 0.01) as there were 11 disagreements. Majority (81.8%) of the disagreements in BIO score grading were within 1-step, while 18.2% were within 2-steps.

## 4 Discussion

To the best of our knowledge, this is the first study assessing the use of SS-OCT as a pre-operative adjunct for vitrectomy for ADED. SS-OCT was able to provide a usable view of the retina in most patients, despite vitreous haze obscuring fundus details on slit lamp examination and fundus photos. It is important to note that at the highest BIO score (5), 50% of the patients had an inadequate view of the fundus on SS-OCT. However, among these 3 patients, 2 had cataracts which may have contributed to the poor images.

The BIO score is a subjective method of evaluating vitreous haze on fundus photos. Consequently there was only moderate intergrader agreement for the BIO score as compared to substantial and almost perfect agreement for fundus photo and SS-OCT diagnosis respectively. An objective method to determine vitreous haze would be ideal to improve reliability. However, to the best of our knowledge, no objective method to measure vitreous haze on fundus photos has been published yet.

Comprehensive pre-operative planning is the cornerstone of performing a successful surgery. A surgeon who has no view of the fundus on examination and imaging when planning for a vitrectomy is more likely to achieve a suboptimal outcome. SS-OCT granting a usable view of the retina in such scenarios can provide the surgeon with information which may alter their approach. In patients with dense VH, having prior information about areas of TRD may help the surgeon choose less risky areas to start the vitrectomy until the retinal view is clear. If patients with ADED are found to have another concurrent pathology such as ERM or macular hole, these pathologies can be treated during the same vitrectomy. In our sample, 4 patients were found to have ERMs and 1 patient had a macular hole. Alternatively, patients who are unlikely to experience much visual improvement, such as due to severe age-related macular degeneration or glaucoma, may be advised not to undergo vitrectomy. In the current era, expectations of patients for post-operative visual outcomes are very high. It is essential to adjust patient expectations by counselling them regarding their visual potential and prognosis. OCT images of outer retinal microarchitecture have been shown to be crucial in determining post-operative BCVA in patients undergoing vitrectomy (13). Similarly, as high wavelength OCT allows for a view of the retina in cases where there is poor view on examination, any hidden pathologies which may affect post-operative visual outcomes can be identified.

Pre-operative OCT has been proven to be a cost-effective measure in patients considering premium intraocular lens implantation (14). In patients undergoing vitrectomy for ADED, pre-operative SS-OCT is a cost-effective measure as it allows for better planning and detection of other pathologies. Improved planning decreases the probability of the patient requiring multiple surgeries, which is likely to avoid a significant cost. Detection of previously undetected ERMs or macular holes could result in resolution of these pathologies during the same surgery.

SS-OCT has facilitated greater understanding of the disease progression of DR as it allows better visualisation of the choroid and vitreous (15). It has been noted that eyes with more advanced stages of DR, such as PDR, are associated with decreased choroidal thickness and vascular density (16, 17). Additionally, diabetic eyes with DR have been shown to have stronger vitreomacular adhesion than other diabetic eyes and the general population, resulting in later onset of posterior vitreous detachment (18). Ishibashi et al. observed that disorganisation of retinal inner layers pre-operatively was associated with poor post-operative BCVA (19). These are important features which further our understanding of DR prognostic factors. Better understanding of the disease process may encourage innovative and novel therapies to counter the growing burden of DR.

It is important to acknowledge the limitations of our pilot study. Firstly, we fell short of our estimated sample size despite a 5-year data collection period, which may impact the generalisability of our results. This was because vitrectomies for ADED are not performed very frequently. Secondly, there is variation amongst imaging parameters between different SS-OCT machines so our results may not be generalisable to other devices. Moreover, the single-centre design of our study and imaging being technician-dependant may potentially introduce bias. Further control studies are required to evaluate the utility of pre-operative SS-OCT in patients undergoing vitrectomy for ADED.

## 5 Conclusion

SS-OCT showed potential as an adjunct to clinical examinations, especially in cases where hazy media opacities did not allow for a clear view of the fundus. It resulted in more comprehensive pre-operative planning, promoting good surgical outcomes. Additionally, it played an important role in guiding patient counselling and assisted in managing their expectations. Despite its potential, it is important to recognise that OCT only serves as an adjunct and cannot be used as a definitive argument for assessing surgical feasibility.

## Data Availability

The raw data supporting the conclusions of this article will be made available by the authors, without undue reservation.
